# Tracing anthropogenic inputs in stream foods webs with stable carbon and nitrogen isotope systematics along an agricultural gradient

**DOI:** 10.1371/journal.pone.0200312

**Published:** 2018-07-06

**Authors:** Kern Y. Lee, Lisa Graham, Daniel E. Spooner, Marguerite A. Xenopoulos

**Affiliations:** 1 Department of Biology, Trent University, Peterborough, ON, Canada; 2 Environmental and Life Sciences Graduate Program, Trent University, Peterborough, ON, Canada; 3 United States Geological Survey, Leetown Science Center, Wellsboro, Pennsylvania, United States of America; University of Hyogo, JAPAN

## Abstract

Stable carbon (^13^C) and nitrogen isotopes (^15^N) are useful tools in determining the presence of agricultural influences in freshwater ecosystems. Here we examined *δ*^15^N and *δ*^13^C signatures in nitrate, fish, and mussel tissues, from rivers in Southern Ontario, Canada, that vary in their catchment proportion of agriculture land use, nutrients and organic matter quality. We found comparatively ^15^N-enriched *δ*^15^N values in animal tissues and dissolved nitrates, relative to expected values characterized by natural sources. We also observed a strong, positive correlation between riparian agriculture percentages and *δ*^15^N values in animal tissues and nitrates, indicating a significant influence of agricultural land use and the probable dominance of organic fertilizer and manure inputs in particular. The use of a ^15^N-based equation for the estimation of fish trophic position confirmed dietary analyses is showing all fish species to be tertiary consumers, with a relatively consistent ^15^N-enrichment in animal tissues between trophic levels. This indicates that variability in ^15^N-trophic fractionation is minor, and that fish and mussel tissue *δ*^15^N values are largely representative of source nitrogen. However, the trophic fractionation value varied from accepted literature values, suggesting strong influences from either local environmental conditions or dietary variation. The *δ*^13^C datasets did not correlate with riparian agriculture, and animal *δ*^13^C signatures in their tissues are consistent with terrestrial C3 vegetation, suggesting the dominance of allochthonous DOC sources. We found that changes in water chemistry and dissolved organic matter quality brought about by agricultural inputs were clearly expressed in the *δ*^15^N signatures of animal tissues from all trophic levels. As such, this study confirmed the source of anthropogenic nitrogen in the studied watersheds, and demonstrated that this agriculturally-derived nitrogen could be traced with *δ*^15^N signatures through successive trophic levels in local aquatic food webs. The *δ*^13^C data was less diagnostic of local agriculture, due to the more complex interplay of carbon cycling and environmental conditions.

## Introduction

Agricultural land use can significantly impact the health and functioning of aquatic ecosystems through increased inputs of organic material, use of organic and chemical fertilizers, and downstream eutrophication [1, 2]. Agricultural fertilizers can often be traced chemically through food webs, given knowledge of uptake pathways and mechanisms of chemical processing, as the signatures of these inputs are transferred from baseline primary producers to higher-level consumers. As such, stable isotopes provide a particularly useful tool to track the presence of agricultural nutrients in local food webs.

Stable nitrogen and carbon isotopes (^15^N and ^13^C) can be traced across successive trophic levels, allowing for the elucidation of potentially cascading influences of human land use through aquatic ecosystems [3, 4]. A number of studies have utilized *δ*^13^C and *δ*^15^N data to investigate the movement of nitrogen and carbon through food chains [5–9], or used such data to discern agricultural influences in the *δ*^15^N signatures of aquatic biota [3, 4, 10, 11]. Many of these have compared the tissue stable isotope signatures of animals in higher trophic levels to those obtained from baseline consumers, in order to investigate the movement of nitrogen and carbon through food chains.

In aquatic systems dominated by anthropogenic influences, strong correlations are typically found between the *δ*^15^N signatures of tissues from both primary and secondary consumers, with agricultural variables such as land-use percentages and fertilizer and manure nitrogen loadings [4, 12]. Other research showed that these agricultural influences could also be traced upwards through successive trophic levels. For example, *δ*^15^N values from primary consumers are tracked to higher-level consumers with a consistent isotope fractionation of about 3.4‰ or less between each level [9]. This ^15^N-fractionation of 3.4‰ has since been considered as a relatively consistent and reliable value that has been applied to subsequent studies.

Past work has also tried to clarify the influences of agriculture on the *δ*^13^C signatures of biota, and have attempted to trace these influence through aquatic food webs. This has proven more difficult as the *δ*^13^C of organic fertilizers and manure are indistinguishable from those of natural or cropland plant material, since the latter is often the primary source of the former. Previous work has instead attempted to differentiate between terrestrial and autochthonous carbon sources within river waters, given that aquatic primary producers often have comparatively lower *δ*^13^C values as compared to those derived from terrestrial soils or plant matter. While attempts have also been made to quantify trophic fractionation of ^13^C across multiple trophic levels, these have encountered far more variation than that observed with ^15^N. For example, a study by Post found *δ*^13^C fractionation values averaging around 0.4 ± 1.3‰ between successive trophic level [9].

This wide range of values may be due to variation within animal species in the same locations. This was found by Hesslein et al. [13], which measured *δ*^13^C differences of up to 7‰ between individuals of the same Lake Trout species, with trophic fractionation values that appeared to be contrary to the expected trend: trout species were found to have lower *δ*^13^C values than white suckers, which should occupy a lower trophic level. Clearly, the trophic fractionation behavior of *δ*^13^C in aquatic ecosystems remains uncertain, and there exists a knowledge gap that needs to be addressed.

A number of studies have utilized *δ*^13^C and *δ*^15^N data to investigate the movement of nitrogen and carbon through food chains[5–9], or used such data to discern agricultural influences in the *δ*^15^N signatures of aquatic biota[3, 4, 10, 11]. Many of these have compared the tissue stable isotope signatures of animals in higher trophic levels to those obtained from baseline consumers and dissolved nitrates, in order to investigate the movement of nitrogen and carbon through food chains [9]. However, few of these previous studies directly linked land use to ^15^N-fractionation in nitrate and food webs, or examined both *δ*^13^C and *δ*^15^N along a gradient of agricultural land use. Even fewer have been truly comprehensive by not only comparing *δ*^15^N and *δ*^13^C data from consumers in two trophic levels, but also including data from primary producers and dissolved nitrate. Here we address this paucity of information by analyzing the *δ*^13^C and *δ*^15^N of dissolved nitrate, periphyton, mussel and fish tissue to provide a comprehensive study of trophic isotope fractionation and the effects of an agricultural land use gradient on isotope systematics.

In this work, we assessed how accurately *δ*^13^C and *δ*^15^N signatures in primary producers and consumers at higher trophic levels reflect the nature of agricultural inputs into aquatic food webs, and how these values vary with an agriculture land use gradient. As well, this study determined if the isotopic fractionation at each trophic level (including between primary producers and primary consumers) is truly consistent. Concurrently, the results will trace the dominant source of anthropogenic nitrogen and carbon in the studied watersheds, and confirm if higher trophic levels are indeed effective indicators of such human-derived nutrient inputs.

## Methods

### Study sites

**Fig 1:**
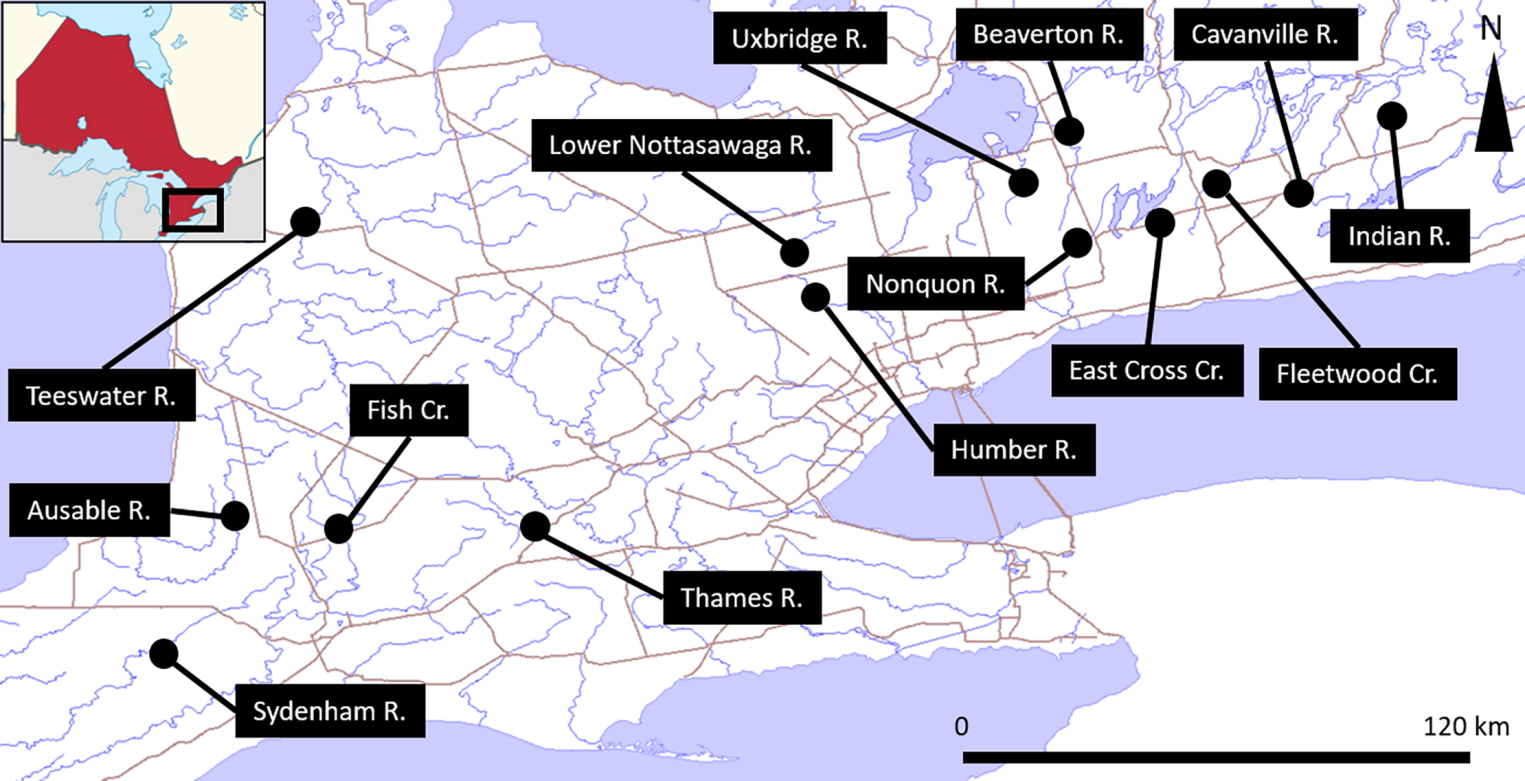
Map of all sampling locations. Basemap data from the Ontario Government, via ESRI Canada.

Fourteen streams in Southern Ontario, Canada, were sampled for water chemistry, mussels, fish and stable isotopes (Fig 1). These streams range in the proportion of cropland in their catchment and have been previously studied to investigate the effects of agriculture on a range of ecosystem responses (e.g., [14–17]). Each watershed varies in catchment area and degree of agriculture land use, with total riparian monoculture (row cropping) making up 8% to 50% of basin surface areas, total catchment cropland making up 18% to 59% of catchment areas, and total catchment areas ranging from 2.9 to 498 km^2^ [18]. The remainder of the land cover is mostly comprised of woodland and wetlands. Corn is the dominant crop type in each basin, with barley, canola, alfalfa, oats, soybeans, wheat, and other legumes present in lesser quantities [19].

### Sample collection

Water and animal tissue samples were collected from each stream under baseflow conditions over the period of August 8 to 17 of 2012. We selected this timeframe to avoid confounding effects of lagged isotopic signals from spring fertilizer applications and novel dietary changes associated with Rock Bass, Brook Trout, and Brown Trout spawning. All sampling occurred on waterways accessed through public access points and no prior permission was required. At each location, 20 L of water were collected at the stream thalweg and stored on ice for later laboratory processing for nutrient and carbon analyses. Site-specific water quality was assessed using an HQ30d portable oxygen and temperature meter and YSI-30 conductivity meter.

Fish were collected using an LR-24 backpack electroshocker and dip nets, in accordance with the Ontario Stream Assessment Protocol ([20], permit MNR 51943). Upon capture, all fish were measured for length and weight, and classified as the following species: Johnny Darters (*Etheostoma nigrum*), Greenside Darters (*Etheostoma blennioides*), Rock Bass (*Ambloplites rupestris*), Brook Trout (*Salvelinus fontinalis*), and Brown Trout (*Salmo trutta*) (the latter three being grouped as “large fish”). Four individuals of 1 to 3 species per stream were euthanized via the application of blunt force in order to avoid tissue contamination associated with the use of chemical agents. The euthanization method was approved by the Trent University Animal Care Committee (ACC), following the submission of an Animal Care Protocol Application. All specimens were later frozen for isotopic analyses of muscle tissue. Gut contents of four to five individuals of bass and trout species from each site were examined to identify dominant food sources. Smaller-sized darters were too small for such an examination (the bulk of the tissue being required for isotopic analyses).

We focused our study on mussels of two common species, *Elliptio complanata* and *Lasmigona costata*. These mussels were located based on site recommendations from a previous 2008 collection as in Spooner et al. (2013) [18, 21]. All field staff received intensive training to recognize all local mussel species, including those listed as threatened, endangered, or at-risk as per Ontario’s Endangered Species Act. If a listed species was found, field staff was instructed to refrain from picking it up and GPS coordinates were reported to the appropriate authorities. If mussels of species *Elliptio complanata* and *Lasmigona costata* were available in the stream, four of each species were collected by hand and placed on ice prior to transport to the lab. Once in the lab, mussels were dissected to obtain a sample of mantle tissue and were subsequently frozen prior to isotope analyses.

In addition, for each mussel sampled, a nearby rock within 0.5 to 1 m distance was randomly selected for periphyton samples either upstream or downstream of the mussel. Both the mussel shells and rock samples were placed into Ziploc bags filled with 125 ml of distilled water before being gently scrubbed of all surface material. The resulting slurries were washed with diluted hydrochloric acid to remove carbonate, before being passed through a GF/F filter (size 0.7 μm) to collect periphyton for stable isotope determinations.

### Laboratory and stable isotope analyses

We analyzed the water samples for dissolved nitrate *δ*^15^N, dissolved organic carbon (DOC) concentrations, total dissolved nitrogen (TDN), and total dissolved phosphorus (TDP). Water collected in the field was immediately filtered upon arrival to the lab through a 0.22 μm pre-rinsed polycarbonate filter for DOC, TDN and TDP analyses. TDN and TDP were both determined by colorimetry with a Varian Cary 50 Bio UV/Visible Spectrophotometer [14]. Water for *δ*^15^N_NO3_ measurements was prepared using the LINX II protocol [22].

All samples were filtered through a 0.2 μm filter, with the dissolved nitrate being converted to ammonium and diffused onto an acidified filter for *δ*^15^N analyses. The instrument was calibrated with reference material USGS40 for all *δ*^15^N and *δ*^13^C measurements every 20 samples, with an internal standard DG (A1) being used for data normalization. All stable isotope samples were analyzed with an EuroEA3028-HTEuroVector Elemental Analyzer (EuroVector SpA, Milan, Italy) coupled with a Micromass IsoPrime Continuous Flow Isotope Ratio Mass Spectrometer (Micromass, UK, with USGS40 standards *δ*^15^N = 4.56‰; *δ*^13^C = 26.39‰) [18]. Standard deviations for all *δ*^15^N and *δ*^13^C measurements were < 0.5‰.

The same instrumentation was utilized for animal tissue (mussels and fish) and periphyton *δ*^15^N and *δ*^13^C analyses, following the method of [23], prior to which samples were dried and ground into powder. Dissolved organic carbon (DOC) concentrations were measured with an OI Analytical 1030D Total Organic Carbon analyzer.

### Stable isotope fractionation estimates

In order to determine the extent to which variability in trophic ^15^N-fractionation could affect fish tissue values, and whether such variation is strong between the sites of study, we estimated trophic ^15^N-fractionation values at all sites with the following formula modified from [7]:

$$\Delta_{n}=\frac{\delta^{15}N_{secondary\;consumer}-\delta^{15}N_{base}}{Trophic\;Level-\lambda }$$ (1)

In Eq (1), “*λ*” represents the trophic position of the organism used to determine “*δ*^15^N_base_” (i.e., the baseline trophic *δ*^15^N value) and “*Δ*_*n*_” is the assumed ^15^N enrichment between each trophic level. The *δ*^15^N values of mussel tissue were used for *δ*^15^N_base_, with the corresponding *λ* being 2 (i.e., primary consumers). Based on the observed fish gut contents, which were exclusively composed of invertebrate species and devoid of smaller fish types in the bass and trout samples, we assumed that all fish were tertiary consumers and a trophic level of “3” was used.

### Statistical analyses

TDN, TDP, and DOC did not meet normality assumptions, and were therefore log-transformed prior to statistical analyses. Across all study sites, we first performed principal component analyses (PCA) using the R statistical software suite (R Development Core Team) to reduce our exploratory dataset [24] and discern any relationships that may exist between a number of key biological and chemical parameters. The PCA we performed used varimax rotation, which results in a new set of components known as varifactors (VF). The correlations between the input variables and the varifactors (termed “loadings”) can then be used to determine significant relationships amongst multiple parameters of interest.

Following [25], we first applied a PCA to land use/land cover percentages (LULC; wetlands, woods, cultivation, developed land, and miscellaneous) in order to integrate all land-use data into a single PCA axis for comparison to all other variables. From this PCA, the VF loadings thought to correspond to agricultural land-use was extracted. These were then incorporated into a second PCA incorporating physico-chemical variables such as TDN, TDP, water temperature, conductivity, dissolved oxygen (DO), *δ*^13^C signatures of fish tissues, and the *δ*^15^N signatures of nitrate and all fish tissues and periphyton (mussel stable isotope data was omitted due to a lack of completeness for a feasible PCA).

In both PCA analyses, variable loadings of 0.5 and higher were considered significant while those lower than 0.5 were assumed to be insignificant and were omitted from discussion (following [26]). We subsequently performed univariate linear regressions of physicochemical data against the land-use VF loadings based on the results of the LULC PCA.

## Results

**Fig 2:**
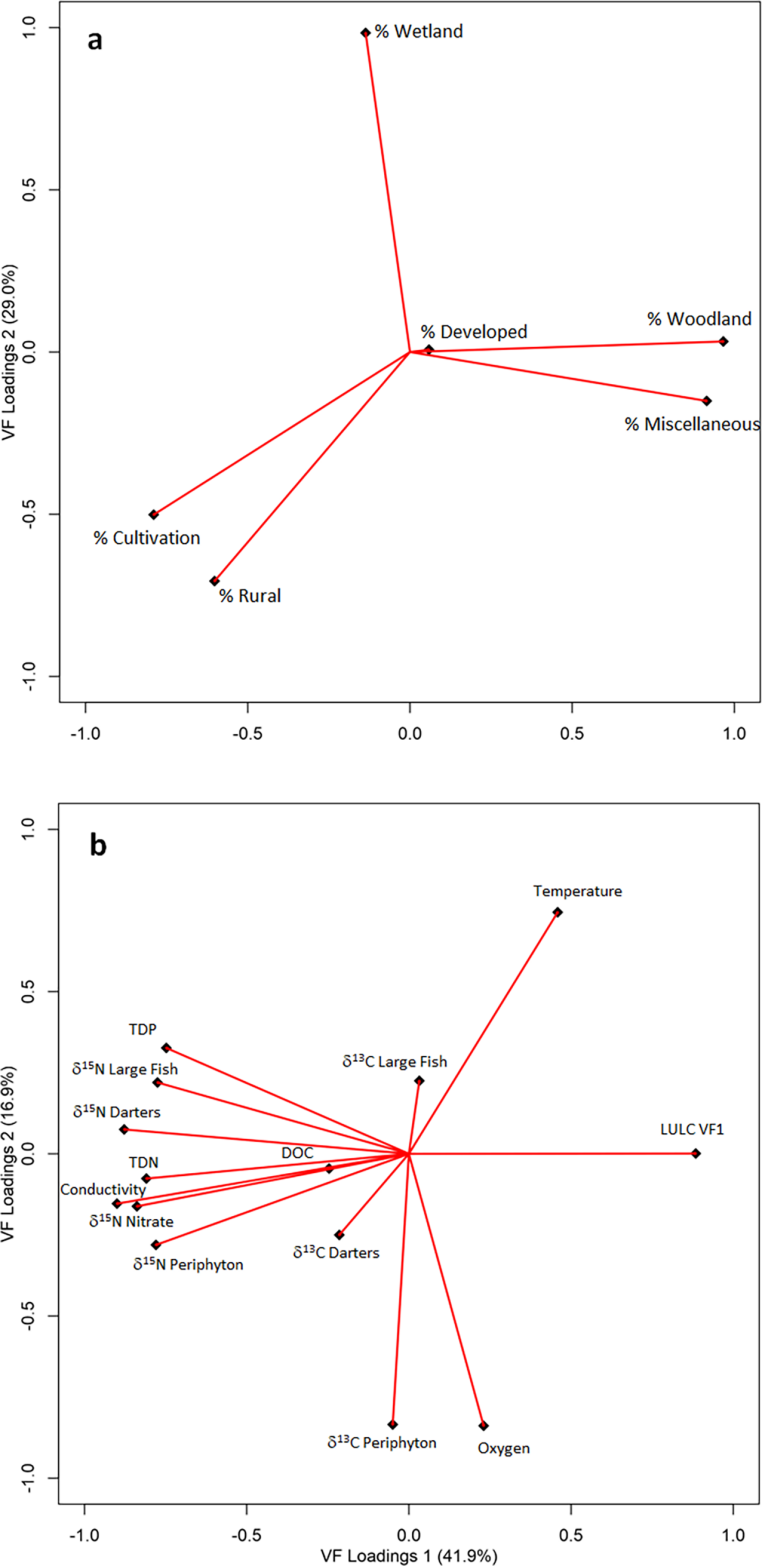
PCA plots utilizing LULC data (a) and physico-chemical data (b).

We extracted 2 varifactors with the LULC PCA analysis, corresponding to 75% of the total variance. Based on this determination, the first varifactor (46.3% of total variance) included strong positive loadings by % cultivation and % rural land, along with a strongly negative loading by % woodland and % miscellaneous (Table 1, Fig 2A). This association suggests a strong association of the first varifactor (LULC VF1) with agricultural land use. Two varifactors were also extracted from the second PCA (with incorporation of LULC VF1 as a variable), which accounted for 58.8% of total variance (Fig 2B). In the first varifactor (41.9% of total variance), strong positive loadings by TDP, TDN, conductivity and all *δ*^15^N datasets were associated with a significantly negative loading by LULC VF1 (Table 1; Fig 2B). The strong relationship between LULC VF1 and the *δ*^15^N datasets with TDN, TDP, and conductivity is indicative of the influence of agricultural land-use.

**Table 1:** Significant PCA results from both the initial LULC PCA and follow-up physico-chemical PCA (with LULC VF1 as a parameter) on selected site parameters.

	Variables	VF1	VF2
LULC PCA	% Woodland	0.97	
	% Wetland	-0.14	0.98
	% Cultivation	-0.79	-0.50
	% Developed		
	% Rural	-0.60	-0.71
	% Miscellaneous	0.92	-0.15
Physco-chemical PCA	TDN	-0.81	
	TDP	-0.75	0.33
	DOC	-0.25	
	Temperature	0.46	0.75
	Conductivity	-0.90	-0.15
	Oxygen	0.23	-0.84
	*δ* ^15^N Nitrate	-0.84	-0.16
	*δ* ^13^C Darters	-0.21	-0.25
	*δ* ^13^C Large Fish		0.23
	*δ* ^15^N Darters	-0.88	
	*δ* ^15^N Large Fish	-0.78	0.22
	*δ* ^15^N Periphyton	-0.78	-0.28
	*δ* ^13^C Periphyton		-0.84
	LULC VF1	0.88	

**Fig 3:**
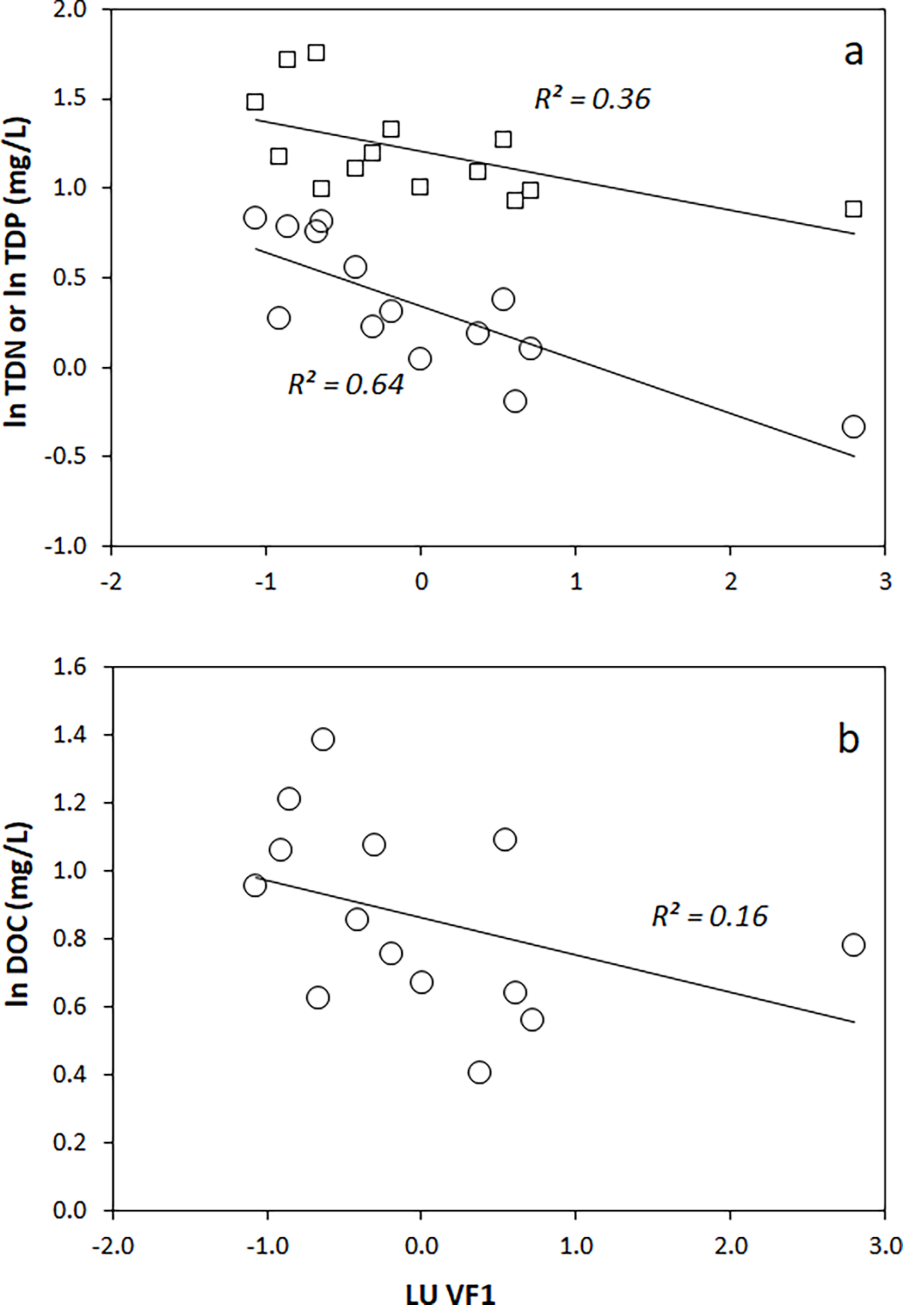
TDN, TDP (a) and DOC (b) plotted against LULC VF1.

Based on the PCA groupings, we then performed univariate regressions comparing LULC VF1, and TDN, TDP, DOC as predictors of *δ*^15^N of nitrate, periphyton and all animal tissues. TDN concentration, which varied from 0.47 to 6.79 mg/L, was significantly correlated with LULC VF1 (*R*^*2*^ = 0.64, *P* < 0.01) (Fig 3A). TDP values, ranging from 7.6 to 56.9 mg/L, showed a positive correlation with riparian agriculture percentages as well (*R*^*2*^ = 0.36, *P* < 0.02) (Fig 3A). DOC ranged from 2.5 to 24.3 mg/L, and was not significantly correlated with LULC VF1 (*R*^*2*^ = 0.16, *P* < 0.05) (Fig 3B).

**Fig 4:**
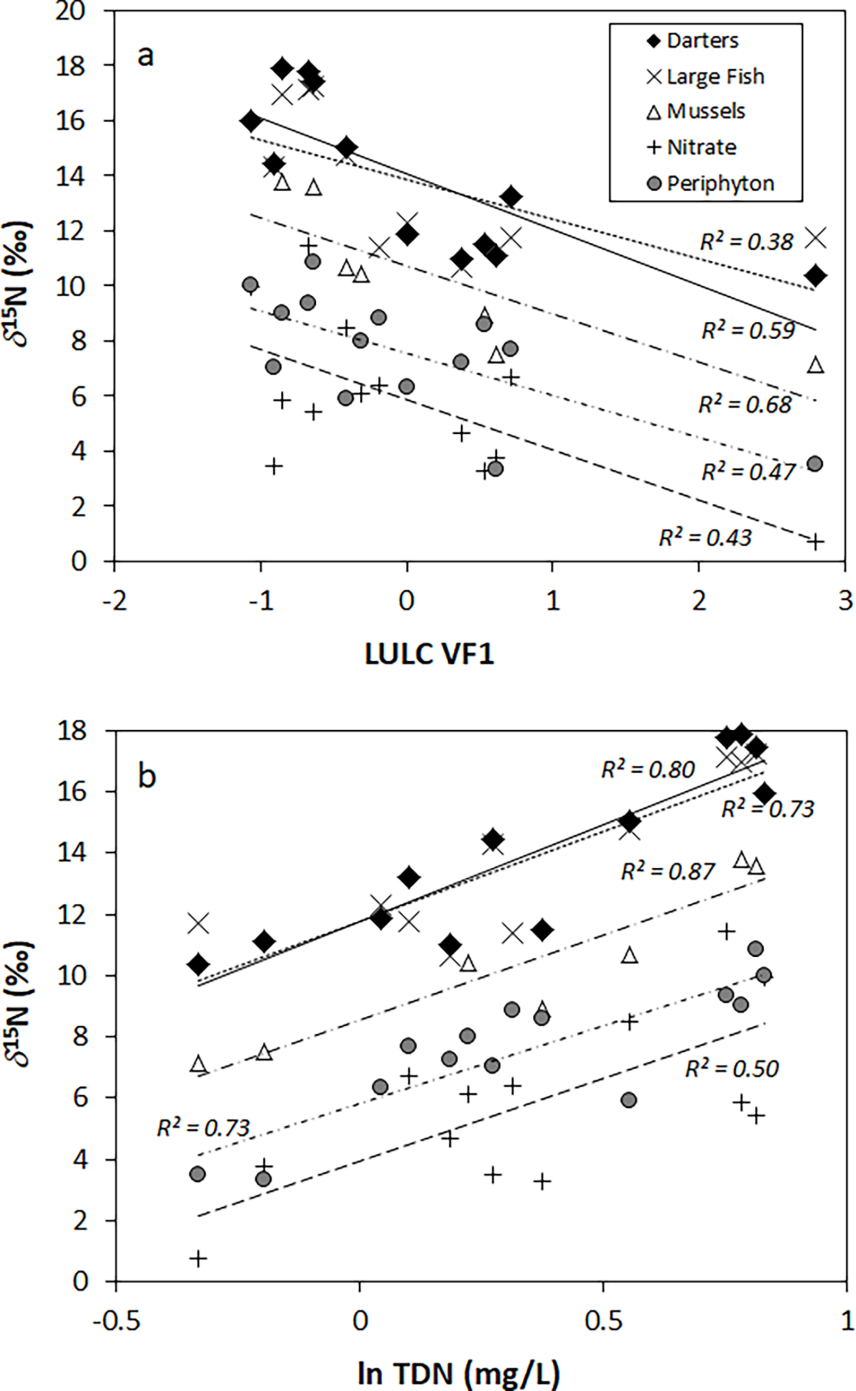
*δ* **^15^N values of nitrate and animal tissues plotted against percent riparian monoculture (A) and TDN (B)**.

The *δ*^15^N_NO3_ data across all sites ranged from 0.7 to 11.4‰, and correlated strongly with LULC VF1 (*R*^*2*^ = 0.43, *P* < 0.05) (Fig 4A). Nitrate *δ*^15^N values increased relative to the expected range for chemical fertilizers and naturally-occurring nitrate derived from atmospheric deposition or soil nitrification processes, and are more characteristic of nitrate present in sewage or manure (Table 2). Periphyton *δ*^15^N values overlapped strongly with nitrate, and were strongly and positively correlated with LULC VF1 (*R*^*2*^ = 0.47, *P* < 0.05) (Fig 4A).

**Table 2:** Landscape and environmental data from all sites of study.

Location	% Wooded	% Wetland	% Cultivation	% Developed	% Rural	% Miscellaneous	TDN (mg/L)	*δ* ^15^N_NO3_ (‰)	Temperature (°C)	Conductivity (mS/cm)	Dissolved Oxygen (mg/L)	TDP (mg/L)	[DOC] (mg/L)
Ausable River	11.3	10.3	50.2	0.9	20.8	3.7	6.1	5.9	18.4	591.5	9.15	51.58	16.18
Beaverton River	7.5	38.9	11.9	0.7	12.1	3.7	1.9	3.5	25.2	510.5	6.22	14.98	11.58
Cavanville Creek	24.7	28.0	17.1	1.2	14.5	4.2	1.1		20.6	482	12.58	10.15	4.71
East Cross Creek	20.2	31.5	18.5	0.5	7.6	4.5	2.1	6.4	18.7	383.3	8.33	21.18	5.71
Fish Creek	11.8	0.1	43.5	0.5	32.9	5.6	6.5	5.4	18.9	587	11.86	9.74	24.31
Fleetwood Creek	32.4	29.3	17.3	0.4	7.3	5.9	0.6	3.8	17	363.1	9.92	8.39	4.38
Humber River	25.6	26.0	8.7	2.5	13.6	11.2	1.3	6.7	17.7	555.5	10.94	9.68	3.65
Indian River	48.6	10.9	8.2	1.5	4.2	19.8	0.5	0.7	23.8	245.6	10.42	7.64	6.06
Nonquon Creek	28.8	24.5	15.0	0.6	15.5	7.7	2.4	3.3	16.9	380.6	9.24	18.72	12.29
Nottawassaga	27.6	27.4	17.2	0.0	17.0	5.4	1.5	4.7	15.1	393.4	11.12	12.3	2.54
Sydenham River	14.6	6.3	39.4	2.2	27.2	4.3	5.7	11.4	17.2	618	8.96	56.86	4.24
Teeswater	17.4	25.3	20.8	0.6	21.5	3.8	3.6	8.5	18.8	554	10.8	12.88	7.22
Thames River	4.0	34.8	29.9	1.1	18.0	4.2	6.8	9.9	16.7	700.5	8.02	29.67	9.05
Uxbridge Brook	17.8	32.2	13.2	3.6	13.4	5.5	1.7	6.1	21.5	476.7	10.42	15.72	11.93

Tissues from all fish species had more positive ^15^N signatures as compared to nitrate (11 to 17.9‰) (Fig 4A, Table 2), as further indicated by their larger y-intercept values when linear regressions were performed against both LULC VF1 and TDN. Both darters (*R*^*2*^ = 0.68, *P* < 0.01) and large fish (*R*^*2*^ = 0.38, *P* < 0.05) showed *δ*^15^N tissue values that correlated strongly with LULC VF1 (Fig 4A). Mussel tissues exhibited a range of *δ*^15^N values that mostly overlapped with those of nitrate (6.3 to 13.6‰) (Table 3), and these also displayed a significant positive correlation with LULC VF1 (*R*^*2*^ = 0.68, *P* < 0.01) (Fig 4A).

**Table 3:** Averaged animal tissue and periphyton stable isotope data from all sites of study.

Location	*δ* ^15^N in Mussel Tissues (‰)	*δ* ^15^N in Darter Tissues (‰)	*δ* ^15^N in Trout/Bass Tissues (‰)	*δ* ^13^C in Darter Tissues (‰)	*δ* ^13^C in Trout/Bass Tissues (‰)	*δ* ^13^C in Mussel Tissues (‰)	*δ* ^15^N of Periphyton (‰)	*δ* ^13^C of Periphyton (‰)
Ausable River	13.78	17.90	16.95	-25.38	-26.67	-30.92	9.0	-19.31
Beaverton River		14.43	14.31	-28.86	-27.83		7.0	-20.83
Cavanville Creek		11.89	12.32	-27.84	-28.20		6.3	-18.42
East Cross Creek			11.38		-24.79		8.9	-18.88
Fish Creek	13.58	17.45	17.23	-26.36	-26.43	-31.38	10.9	-19.41
Fleetwood Creek	7.50	11.10		-29.00		-28.72	3.3	-15.27
Humber River		13.24	11.74	-27.18	-26.32		7.7	-18.08
Indian River	7.15	10.36	11.73	-27.62	-26.19	-28.13	3.5	-22.26
Nonquon Creek	8.94	11.51		-29.86		-33.96	8.6	-22.88
Nottawassaga		10.99	10.66	-29.18	-29.83		7.2	-18.86
Sydenham River		17.77	17.13	-28.10	-26.79		9.3	-22.49
Teeswater	10.67	15.06	14.75	-27.56	-28.05	-30.82	5.9	-19.02
Thames River		15.97		-28.98			10.0	-20.07
Uxbridge Brook	10.43					-32.38	8.0	-24.89

**Fig 5:**
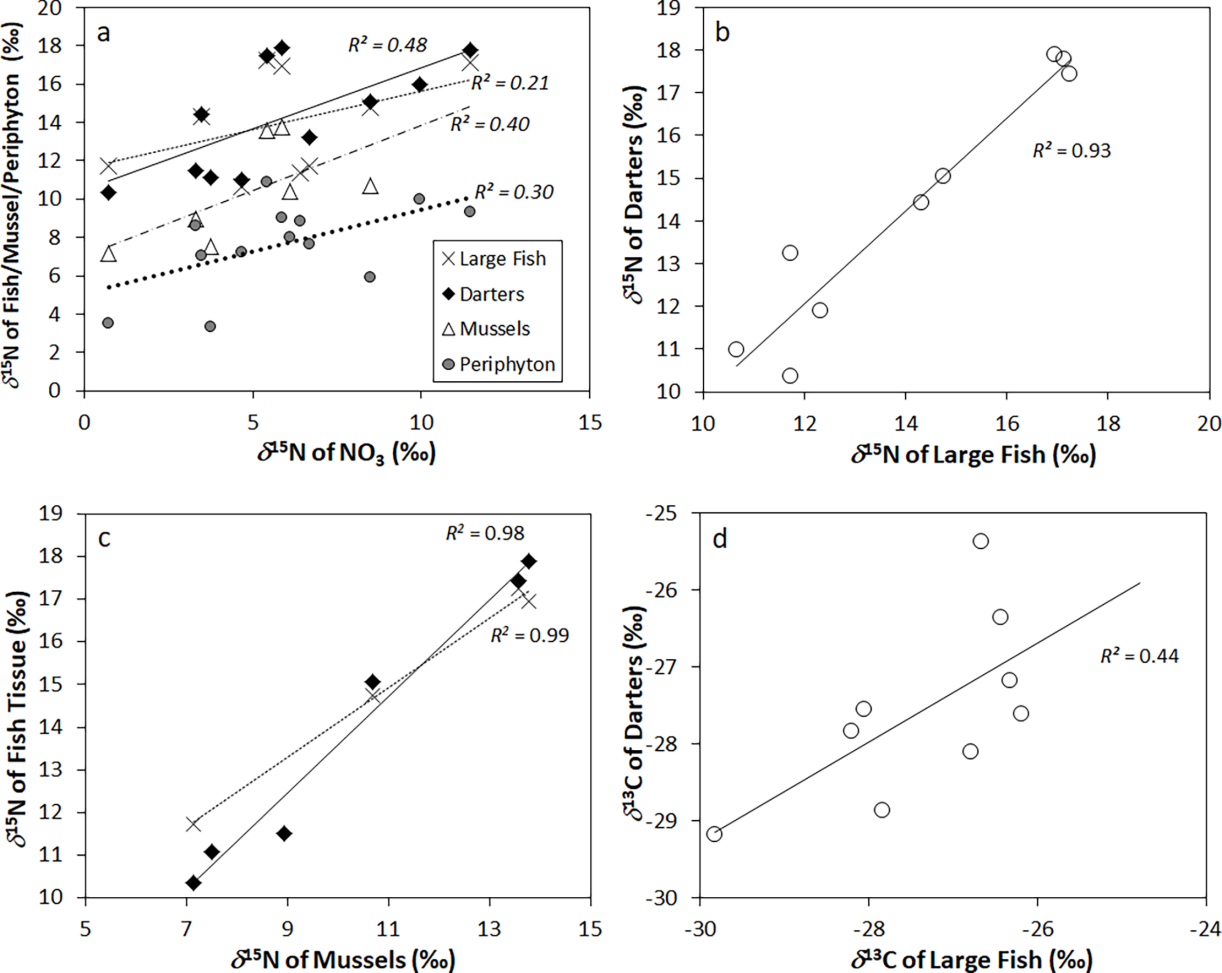
*δ*^15^N of animal tissues plotted against *δ*^15^N of nitrate (a), *δ*^15^N of darters plotted against *δ*^15^N of trout and bass (b), *δ*^15^N of fish tissues plotted against *δ*^15^N of mussles (c), and *δ*^13^C of darters plotted against *δ*^13^C of trout and bass (d).

The strong relationships between all animal *δ*^15^N tissues, *δ*^15^N of periphyton, *δ*^15^N_NO3_, and TDN derived from the second PCA was investigated with an additional series of univariate regressions. TDN correlated strongly and positively with the *δ*^15^N of darters (*R*^*2*^ = 0.80, *P* < 0.01), large fish (*R*^*2*^ = 0.73, *P* < 0.01), and mussel tissue (*R*^*2*^ = 0.89, *P* < 0.01). Correlations of TDN against the *δ*^15^N of nitrate, while statistically significant, were weaker than that of animal tissues (*R*^*2*^ = 0.50, *P* < 0.01) (Fig 4B). However, a positive correlation of the *δ*^15^N of periphyton and TDN was comparatively strong (*R*^*2*^ = 0.72, *P* < 0.01). Mussel *δ*^15^N values displayed a significant positive correlation with the *δ*^15^N_NO3_ data (*R*^*2*^ = 0.75, *P* < 0.01), as did tissue *δ*^15^N from darter tissue *δ*^15^N (*R*^*2*^ = 0.48, *P* < 0.05), and periphyton *δ*^15^N (*R*^*2*^ = 0.43, *P* < 0.01) (Fig 5A).

Correlations between animal tissue *δ*^15^N datasets were also strong. The *δ*^15^N values in darter tissues were significantly related with those in larger fish species (*R*^*2*^ = 0.93, *P* < 0.01) (Fig 5B). Positive correlations between all fish tissue *δ*^15^N signatures and those of mussel tissues were significant as well (*R*^*2*^ = 0.97 and *P* < 0.01 for trout and bass, and *R*^*2*^ = 0.48 and *P* < 0.01 for darters) (Fig 5C).

We also explored correlations between the animal tissue *δ*^13^C datasets. The *δ*^13^C of mussel tissues ranged from -28 to -34‰ and were comparatively ^13^C-depleted relative to all fish types, which exhibited tissue *δ*^13^C values ranging from -25 to -30‰ (Table 2). As with the *δ*^15^N data, the *δ*^13^C values of darter tissues did show a significant correlation with those of bass and trout species (*R*^*2*^ = 0.43, *P* < 0.01) (Fig 5D). Periphyton *δ*^13^C showed no significant correlations with those of fish and mussel tissues.

As well, none of the animal tissue or periphyton *δ*^13^C datasets was significantly related to land use, water temperature, conductivity, or dissolved oxygen. The stomach content analyses showed that bass and trout species were devoid of smaller fish types, and their diets were instead dominated by invertebrates such as crayfish, water beetles (*Gyrinidae*), midges (*Chironomidae*), and nematodes (Table 4).

**Table 4:** The presence or absence of prey items in the stomachs of selected fish species (4 specimens collected at each site).

Site	Examined Fish Species	Crayfish	Gyrinidae	Chironomidae	Nematode	Unidentifiable (Liquid)	Empty
Ausable River	Rock Bass	0	1	0	1	1	1
Fish Creek	Rock Bass	4	0	0	0	0	0
Sydenham River	Rock Bass	3	0	0	0	0	1
Beaverton River	Rock Bass	3	0	0	0	0	1
East Cross Creek	Rock Bass	0	1	0	0	3	0
Teeswater	Rock Bass	3	0	0	0	0	1
Cavanville Creek	Rock Bass	4	0	0	0	0	0
Nottawassaga	Brook Trout	0	0	1	0	2	1
Humber River	Brown Trout	0	1	1	0	1	0
Indian River	Rock Bass	1	0	0	0	1	2

**Fig 6:**
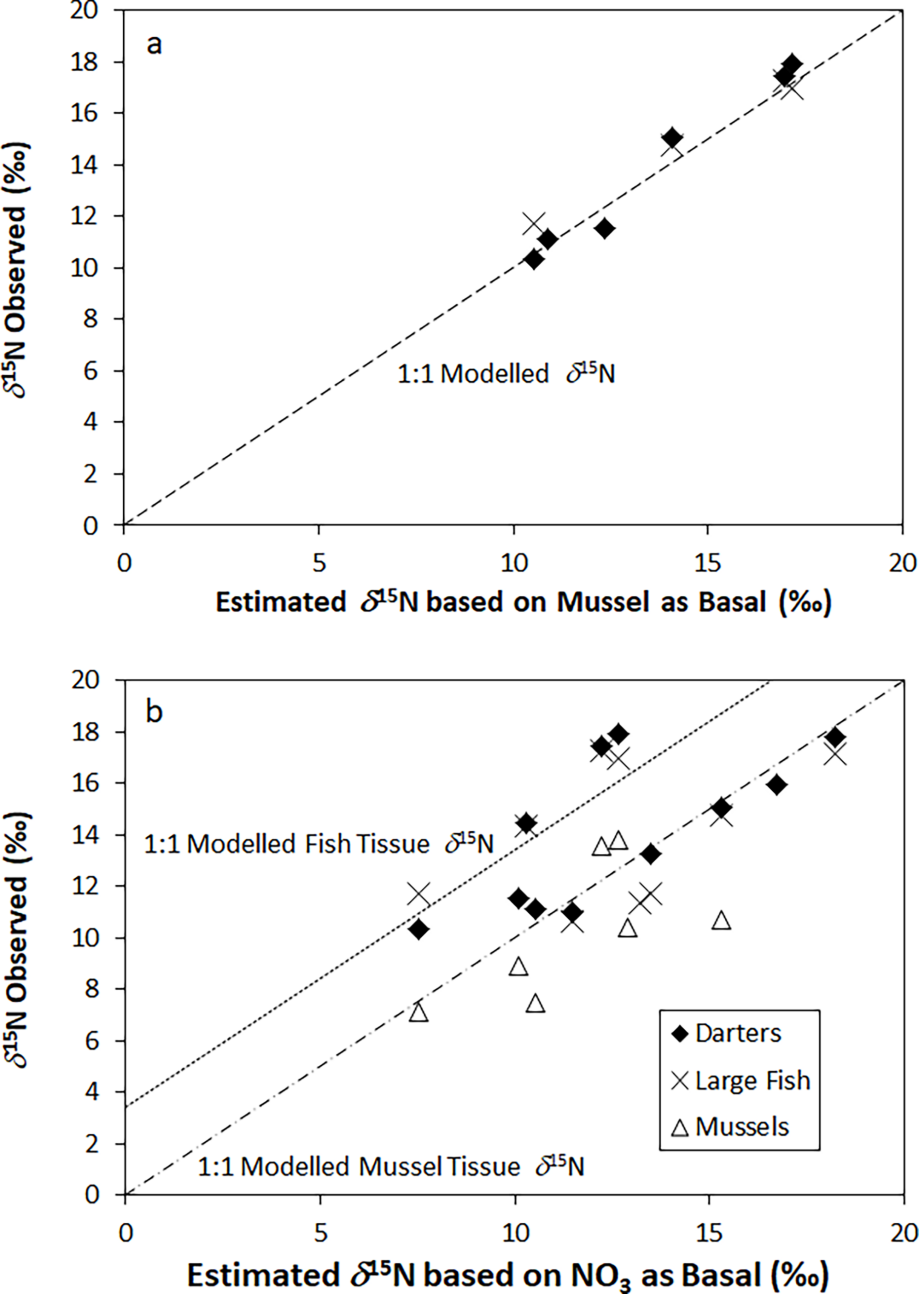
A comparison of observed and modelled animal tissue *δ*^15^N values based on the equation of [17], using an assumed *δ*^15^N of 3.4‰ and using either the *δ*^15^N of nitrate (A) or mussel tissues (B) as baseline values.

Using Eq (1), darters and larger bass and trout species had an average trophic ^15^N-fractionation of 3.90 ± 0.50‰ and 3.87 ± 0.60‰, respectively, which lies within the literature trophic fractionation range of 3 to 4‰. We also observed a close correspondence of collected animal tissue *δ*^15^N data and modelled values, assuming an average fractionation value of 3.4‰ [12] and using either *δ*^15^N_NO3_ or the *δ*^15^N of mussel tissue as trophic baselines (Fig 6A and 6B).

## Discussion

We found that agriculture strongly influences TDN and all *δ*^15^N datasets, and is most clearly reflected in the *δ*^15^N signatures of animal tissues, periphyton, and nitrate. The latter is more ^15^N-enriched than anticipated relative to natural sources, and instead resembles organic fertilizer and manure values which typically range from 7 to 20 ‰ [27]. Our findings indicate that the *δ*^15^N signatures of nitrates, periphyton, and animal tissues from all trophic levels are clear indicators of agricultural influence, since this cropland-derived nitrogen is also reflected in their *δ*^15^N.

A probable explanation for the comparatively ^15^N-enriched *δ*^15^N_NO3_ signatures and their strong positive correlations with agricultural land use is that the dissolved nitrogen at all locations is predominantly sourced from organic fertilizers and/or manure. This possibility is supported by the association of positive loadings by LULC VF1, *δ*^13^N_NO3_, TDP, TDN, conductivity, and *δ*^15^N of all animal tissues and periphyton in the PCA analyses. A similar trend was described by [27], who found that the *δ*^15^N signatures of nitrate in river waters collected in the Northeastern United States showed increasing ^15^N-enrichment with larger inputs of wastewater and manure. As well, studies by Pastor et al. [28] and Peipoch et al. [29] found that anthropogenic NO_3_ consistently exhibited the highest *δ*^15^_NO3_ signatures, with these also being expressed in the *δ*^15^N values of aquatic vegetation.

Processes such as bacterial denitrification and plant nitrate-uptake are also known to enrich residual nitrate in ^15^N [30, 31], and our previous work showing increases in bacterial activity with agriculture land use raises the possibility of such contributions to the observed ^15^N-enrichments [15, 18]. Normally, we would expect a negative correlation between *δ*^15^N_NO3_ and TDN if these denitrifying processes were prevalent, as the utilization of nitrate would reduce their concentrations as isotopic fractionation progressed [31, 32]. However, significant loadings of nitrogen derived from the dominance of denitrification processes could overcome this expected trend ([4, 12, 33]). Therefore, nitrate subject to denitrification could be another source of agriculturally-derived ^15^N-enriched nitrogen, perhaps in addition to fertilizer and manure application.

The positive relationships observed between the *δ*^15^N of mussel tissues and both *δ*^15^N_NO3_ and riparian monoculture percentages is further indicative of a significant agricultural influence on the *δ*^15^N signatures of baseline consumers. Comparable findings were obtained from studies by [10] and [11], which found that the *δ*^15^N of *E*. *complanata* was more enriched in agricultural and developed areas, as compared to forested sites. As well, a study by [4] found strong positive correlations between agricultural land-use percentages and the *δ*^15^N values of primary consumers, predatory invertebrates, and fish. Our study builds on this research by considering primary production in the form of periphyton, which was also shown to correlate with land use and *δ*^15^N_NO3_.

These findings, in addition to the strong correlations between mussel and fish tissue *δ*^15^N values, validate the utility of mussels for use as a trophic baseline. We assumed that the more enriched *δ*^15^N values in mussel tissues relative to nitrate, and in fish tissues relative to mussels, were primarily the result of trophic fractionation. This assumption is supported by the estimated ^15^N trophic fractionation values, which appear to broadly agree with those cited in earlier studies although some stronger variation occasionally exists.

However, the use of mussel tissue as a baseline appears to show a stronger fit between modelled and observed *δ*^15^N fish tissue signatures as compared to nitrate, which suggests a weaker correspondence between mussel tissue and nitrate. This is unexpected, as relationships between primary consumers and terrestrially-derived nitrate would likely be stronger than those between dissolved nitrate and secondary (or tertiary) consumers. The weaker correspondence could be a reflection of the diverse diet of mussels, which can include phytoplankton, zooplankton, bacteria, and general organic detritus derived from both aquatic and terrestrial sources [29]. All of these inputs vary in their *δ*^15^N signatures, which would lead to stronger variability in mussel tissue *δ*^15^N signatures and a weaker dependence of these on *δ*^15^N_NO3_.

In turn, the fish *δ*^15^N signatures are expected to correlate more strongly with those of mussel tissues, as mussel diets are generally representative of the bulk organic material in a given area [11]. Nevertheless, the correlation between mussel tissue *δ*^15^N and that of dissolved nitrate remains strong, which may be due to the influence of organic fertilizers on terrestrial organic matter [27]. If mussel diets are composed of appreciably large amounts of terrestrially-derived organic material, which in turn may incorporate a significant amount of fertilizer-sourced nitrogen, we would expect such a correlation between mussel tissue and dissolved nitrate.

Dietary variation may also account for the larger *δ*^15^N trophic fractionation values in all fish tissues as compared to the often-cited value of 3.4‰ [9]. Darters and larger species at some locations showed fractionation values as high as 4.38‰ and 4.58‰, respectively. Some of the trout specimens were found to contain whirligig beetles (*gyrinidae*), which are tertiary consumers and therefore are likely to be more ^15^N-enriched as compared to secondary consumers such as crayfish, midgefly larvae, or nematodes. The incorporation of *gyrinidae* into trout diets could shift tissue *δ*^15^N signatures towards more ^15^N-enriched values.

However, the *δ*^15^N values in darters are comparable to, and usually indistinguishable from, those observed in trout/bass. As the latter are expected to occupy a higher trophic level, one would normally expect the *δ*^15^N of darter tissues to be lower as compared to larger fish species. Previous studies have shown both greenside and johnny darters to incorporate similar prey, such as midgefly larvae and chironomids in their diet [34–37]. So rather than dietary variation, the convergence of *δ*^15^N darter and trout/bass values may instead suggest a genuine deviation from the expected 3.4‰ trophic fractionation value proposed by Post [9], although this difference is nonetheless comparatively minor.

Alternatively, the use of mussels as proxies for trophic consumer baseline values may not be entirely appropriate as a comparison to small and large fishes, with aquatic invertebrates (i.e., the preferred prey of all fish types) perhaps being more suitable for trophic ^15^N-fractionation estimates. Nonetheless, this discrepancy in ^15^N fractionation values highlights the possibility of variation in trophic isotope fractionation that is dependent on the studied region, and raises questions about the blind acceptance of a consistent trophic fractionation across all locations and environments.

In contrast to the *δ*^15^N data, the influence of land use on the *δ*^13^C signatures of animal tissues and periphyton is uncertain, as agricultural inputs would largely depend on the vegetation type comprising the DOC. Although corn, a C4 plant with a comparatively more ^13^C-enriched signature (~-14‰) as compared to C3 (~-26‰) vegetation [38], dominates much of the basin, a significant portion of riverine DOC is likely derived from fertilizer sourced from animal manure (as with nitrogen inputs). These would likely be comprised predominantly of C3 vegetation-derived soil carbonates and organic material that would be indistinguishable from natural sources, given the grass-based diet of cattle.

Our averaged *δ*^13^C values within fish tissues are generally consistent with median terrestrial C3 vegetation values. This is likely an expression of fish diet, as the observed stomach contents show that secondary consumers that feed largely on terrestrial detritus comprise the bulk of the fish diets [36]. On average, individual mussel tissue *δ*^13^C values are more ^13^C-depleted as compared to either darter-type species or trout and bass. However, this relative depletion is inconsistent, with average *δ*^13^C differences between mussel and fish tissues of 3.76 ± 3.02‰ and 0.14 ± 1.43‰ for darter-type species and bass/trout, respectively.

This comparative *δ*^13^C-depletion, and lack of correlation between fish and mussel *δ*^13^C tissue values, is likely due to a strong sensitivity of *δ*^13^C signatures to dietary differences. As mentioned previously, mussels are omnivorous and their food sources vary depending on their habitat, which likely results in correspondingly strong variations in the *δ*^13^C of food sources [37]. For example, mussels may filter-feed on zooplankton and phytoplankton from the open water column in streams, interstitially from the sediment, or pedal feed on bulk fractions of organic matter in sediment [21, 34].

These local habitat differences are more likely to affect mussel *δ*^13^C values rather than those of fish, which are consistently representative of organic material and biota within the water column. Therefore, in contrast to the ^15^N datasets, the mussel data may be less reliable as indicators of baseline trophic ^13^C values. A more accurate surrogate for a trophic *δ*^13^C baseline would likely be the invertebrates that most fish species directly feed on, such as crustaceans and insects.

The larger *δ*^13^C of periphyton relative to fish or mussel tissues also indicates that periphyton may not be an accurate indicator of bulk in-situ primary production *δ*^13^C values. This may be due to a stronger influence of soil-derived carbonates, given the similarity of periphyton *δ*^13^C values to soil-respired carbon (around -23.3‰; [38]). Aquatic algae rather than periphyton would likely provide *δ*^13^C values more representative of in-stream primary production, given that much terrestrially-derived detrital material may be incorporated into the collected periphyton samples.

## Conclusions

We found agriculturally-derived nitrogen to be strongly related to the *δ*^15^N datasets and are comparable to expected organic fertilizer values. However, estimates of fish trophic position through use of *δ*^15^N data show occasionally larger deviations in trophic fractionation estimates from those in the literature. This indicates that caution should be exercised when assuming a uniform trophic isotope fractionation value across all locations of study, and that the use of a single, static value is an oversimplification. It may be more likely that ^15^N fractionation values vary more than previously supposed, and that this variation needs to be taken into account in future studies.

As well, our study shows that care must be taken when selecting an assumed trophic baseline upon which to estimate isotope fractionation values, which may vary widely depending on either the organism type or even its sampled location. In the case of this study, neither mussels nor periphyton may be accurate representatives of primary consumers and primary producers, respectively, and algae and small invertebrates are likely to be more appropriate for such studies. The wide range of ^15^N-signatures in dissolved nitrate also indicates the need to characterize source nitrogen with greater precision through further constraints on isotope data. This may require the *δ*^15^N analysis of other nitrogen species, such as ammonium, or the use of *δ*^18^O measurements.

The ^13^C data is less diagnostic of agricultural influences and show no consistent trophic isotopic fractionation, suggesting that carbon cycling dynamics are more complex as compared to those of nitrogen. Aquatic carbon cycles are composed of a complicated interplay of biological, geological, and even climatic variables, with any of the above possibly being a dominant factor depending on location. As such, ancillary measurements of both organic and inorganic carbon species should be included for additional clarity, in conjunction with more comprehensive sampling of additional biota. In particular, the *δ*^13^C values of aquatic invertebrates, rather than mussel tissue, may serve as a more appropriate trophic baseline in future studies due to their greater importance in fish diets.
